# A self-training subspace clustering algorithm based on adaptive confidence for gene expression data

**DOI:** 10.3389/fgene.2023.1132370

**Published:** 2023-03-21

**Authors:** Dan Li, Hongnan Liang, Pan Qin, Jia Wang

**Affiliations:** ^1^ Faculty of Electronic Information and Electrical Engineering, Dalian University of Technology, Dalian, Liaoning, China; ^2^ Department of Breast Surgery, The Second Hospital of Dalian Medical University, Dalian, Liaoning, China

**Keywords:** self-training, subspace clustering, label confidence, adaptive adjustment, gravitational search algorithm, gene expression data

## Abstract

Gene clustering is one of the important techniques to identify co-expressed gene groups from gene expression data, which provides a powerful tool for investigating functional relationships of genes in biological process. Self-training is a kind of important semi-supervised learning method and has exhibited good performance on gene clustering problem. However, the self-training process inevitably suffers from mislabeling, the accumulation of which will lead to the degradation of semi-supervised learning performance of gene expression data. To solve the problem, this paper proposes a self-training subspace clustering algorithm based on adaptive confidence for gene expression data (SSCAC), which combines the low-rank representation of gene expression data and adaptive adjustment of label confidence to better guide the partition of unlabeled data. The superiority of the proposed SSCAC algorithm is mainly reflected in the following aspects. 1) In order to improve the discriminative property of gene expression data, the low-rank representation with distance penalty is used to mine the potential subspace structure of data. 2) Considering the problem of mislabeling in self-training, a semi-supervised clustering objective function with label confidence is proposed, and a self-training subspace clustering framework is constructed on this basis. 3) In order to mitigate the negative impact of mislabeled data, an adaptive adjustment strategy based on gravitational search algorithm is proposed for label confidence. Compared with a variety of state-of-the-art unsupervised and semi-supervised learning algorithms, the SSCAC algorithm has demonstrated its superiority through extensive experiments on two benchmark gene expression datasets.

## 1 Introduction

The recent development of biological experiments has generated vast amounts of gene expression data. Thus, comprehending and interpreting the enormous number of genes has become a significant challenge (Diniz et al., 2019; Maâtouk et al., 2019; Li and Yang, 2020; Summers et al., 2020; Nisar et al., 2021; Dang et al., 2022). Semi-supervised learning (Chapelle et al., 2006) is a focused issue in the analysis of gene expression data, the research branches mainly include semi-supervised gene clustering (Yu et al., 2014; Yu et al., 2016; Xia et al., 2018; Liu et al., 2021), semi-supervised gene classification (Huang and Feng, 2012; Zhang et al., 2021), semi-supervised gene selection (Mahendran et al., 2020), and semi-supervised gene dimensionality reduction (Feng et al., 2021). In this paper, we focus on the semi-supervised gene clustering problem for identify co-expressed gene groups, which can provide a useful basis for the further investigation of gene function and gene regulation in the field of functional genomics (Maâtouk et al., 2019). When clustering gene expression data, practical dataset usually exists in the form of a large amount of unlabeled data and a small amount of labeled data. However, unsupervised clustering algorithms inherently lack the ability to utilize the label information in exploring the pattern of gene expression data, and the clustering results are often unsatisfactory. Comparatively speaking, semi-supervised clustering can make full use of prior knowledge, such as pairwise information or class labels, to guide the partition of unlabeled data, thus can improve the clustering quality of gene expression data.

Most of the existing semi-supervised learning methods use raw data directly for analysis (Gan et al., 2013; Wu et al., 2018; Li et al., 2019). In recent years, many scholars have found in their research that the intrinsic structure of data is often smaller than its actual dimensionality, and it may be easier to mine the cluster structure of data in subspaces (Basri and Jacobs, 2003). Subspace-based low-dimensional feature representation of data has been successfully applied to various applications, such as image segmentation (Liu et al., 2013; Fei et al., 2017; Xu et al., 2023) and biological data analysis (Shi et al., 2019; Wang et al., 2019; Zheng et al., 2019; Lu et al., 2020; Sun et al., 2021; Huang and Wu, 2022). One of the representative algorithm is low-rank representation (LRR) (Liu et al., 2013), which assumes that the dataset is sampled from multiple mutually orthogonal subspaces in the data space, and uses rank to measure the sparsity of matrix. LRR only focuses on the global structure of data, and ignores the local structure hidden in data. To overcome this drawback, Wang et al. (2019) introduced mixed-norm and Laplacian regularization into LRR to identify differentially expressed genes for tumor clustering. Lu et al. (2020) incorporated the constraints of the non-negative symmetric low-rank matrix and graph regularization for cancer clustering. To preserve the neighbor relationship among data, Fei et al. (2017) proposed a low-rank representation algorithm with distance penalty (LRRADP), which adds a distance penalty term on the basis of LRR to ensure that the representation vectors of the neighboring data in the original data space are still close in the representation space, thereby enhancing the locality of the model and data discriminability. Aiming at guaranteeing block diagonal property of LRR, Xu et al. (2023) presented a projective block diagonal representation approach, which rapidly pursues a representation matrix with block diagonal structure. By assuming that cells with the same type are in the same subspace, Zheng et al. (2019) proposed a self-expression clustering method with non-negative and low-rank constraints for cell type detection. Besides, to effectively integrate multiple omics data, various multi-view subspace clustering algorithms based on LRR were developed for cancer subtyping (Shi et al., 2019; Sun et al., 2021; Huang and Wu, 2022).

As an essential semi-supervised learning method, self-training (Nie et al., 2012; Gan et al., 2013; Wu et al., 2018; Xia et al., 2018; Li et al., 2019) has been successfully applied to various applications including the analysis of gene expression data. Self-training can be regarded as a kind of self-learning method, which consists of two main steps (Li et al., 2019): semi-supervised learning using labeled data to update the predicted labels of unlabeled data; expansion of labeled dataset by selecting unlabeled data as newly labeled data based on some rules. These two steps are repeated until some stopping criteria are reached. For the task of self-training classification, Gan et al. (2013) suggested utilizing unlabeled and labeled data to reveal the true data space structure by cluster analysis, along with a semi-supervised fuzzy c-means technique, to improve self-training. However, the algorithm is not appropriate for non-spherically distributed data (Wu et al., 2018; Li et al., 2019). To overcome this weakness, Wu et al. (2018) proposed a method of self-training based on density peak of data (STDP), which uses clustering by fast search and find of density peaks (DPC) (Rodriguez and Laio, 2014) to build the density-pointing relationship between data, and newly labeled data are selected to iteratively strengthen the classification performance of SVM, KNN, and CART on this basis. Although STDP achieves good classification results for non-spherically distributed data, the problem of mislabeling in the self-training process is not considered. In fact, mislabeling of newly labeled data in a self-training approach is an unavoidable and very intractable problem (Li et al., 2019). Iterative self-training based on these mislabeled data will further reinforce the misinformation and generate more mislabels, leading to mistaken reinforcement (Xia et al., 2018; Li and Zhu, 2020). To solve this problem, researchers have proposed various self-training methods based on partial noise filters in recent years, including multi-label self-training with editing (Wei et al., 2013), dynamic safety assessment self-training based on semi-supervised learning and data editing (Liu et al., 2019), etc. To further exploit unlabeled data in the filter and overcome the parameter dependence problem, Li et al. (2019) proposed a self-training method based on density peaks and an extended parameter-free local noise filter (STDPNF), which can filter out part of mislabeled newly labeled data. However, as with other self-training algorithms using local noise filters, STDPNF still needs to entirely solve the problem of mislabeling.

On the other hand, for the self-training clustering task, Nie et al. (2012) proposed an active self-training clustering (ASTC), which utilizes Gaussian fields and harmonic functions (GFHF) (Zhu et al., 2003) to achieve label propagation. ASTC considers the probability of data being partitioned into various clusters as Bayesian posterior probability, and iteratively selects unlabeled data with large probability values as newly labeled data to optimize the label fitness process of GFHF and improve the label prediction accuracy. To address the problem of partitioning cancer gene expression data, Xia et al. (2018) proposed a self-training subspace clustering algorithm under low-rank representation (SSC-LRR), which introduces LRR to extract subspace structures from cancer gene expression data, iteratively clusters low-rank representation matrix and noise matrix using the K-means algorithm, and selects unlabeled data with the same clustering labels on the two matrices as newly labeled data for self-training learning. SSC-LRR achieves encouraging cancer classification on several benchmark gene expression datasets, and the advantage of low-rank representation in extracting discriminative features from data is analyzed through experimental results.

Despite the success of the above self-training methods, mislabeling a considerable amount of newly labeled data is inevitable (Xia et al., 2018; Li et al., 2019), and its accumulation will lead to the problem of mistaken reinforcement and seriously affect the performance of the self-training methods. In fact, in the self-training clustering problem on gene expression data, different newly labeled data should have different label confidences. The higher the semi-supervised learning value of a newly labeled datum, the more likely this datum has a correctly predicted label, so it should be assigned a higher label confidence. Based on the above analysis, for gene expression data with partial labels, a self-training subspace clustering algorithm based on adaptive confidence (SSCAC) is proposed in this paper, with the following main contributions. Firstly, a self-training subspace clustering framework based on GFHF is designed in this paper, which reveals the subspace structure of gene expression data through low-rank representation, and achieves iterative semi-supervised learning of unlabeled data using the label propagation capability of GFHF on the basis of the constructed similarity matrix. Secondly, to tackle the problem of mislabeling, an improved GFHF objective function with label confidence and the corresponding adaptive adjustment strategy of label confidence based on the gravitational search algorithm (Rashedi et al., 2009) are proposed. The negative impact of mislabeled data can be mitigated by reducing the label confidences of low-value newly labeled data, and the clustering accuracy on gene expression data can thus be improved.

## 2 Proposed algorithm

Although existing self-training methods have improved the partition accuracy of unlabeled data to some extent, the mislabeling problem of newly labeled data is still one of the important challenges in self-training methods (Xia et al., 2018; Li et al., 2019), which makes it difficult to accurately identify co-expressed gene groups on gene expression data with partial labels. During the iterative self-training, the falsely predicted labels will be accumulated gradually and lead to the problem of mistaken reinforcement. One major reason is that once the newly labeled data are selected, self-training methods always fully trust their predicted labels in the semi-supervised classification or clustering process, i.e., it is implicitly assumed that all newly labeled data have the same label confidence. This will obviously make both correctly and incorrectly labeled data act on the semi-supervised learning task with equal strength, and ignore the difference in value of different newly labeled data for semi-supervised learning. In view of this, a self-training subspace clustering algorithm based on adaptive confidence for gene expression data (SSCAC) is proposed in this paper. The proposed algorithm uses density relationships to select newly labeled data, and constructs a self-training subspace clustering framework based on GFHF and the low-rank representation with distance penalty. SSCAC differs from the existing self-training methods in that the semi-supervised clustering objective function with label confidence and the adaptive adjustment strategy of label confidences. The proposed algorithm aims to weaken the supervisory guidance of low-value newly labeled data by reducing their label confidences, thus alleviating the problem of mislabeling in the self-training process and improving the generalization ability of the algorithm.

### 2.1 SSCAC objective function

Currently, low-rank representation has achieved good clustering results as a typical representation model for learning the subspace structure of gene expression data (Xia et al., 2018; Shi et al., 2019; Wang et al., 2019; Zheng et al., 2019; Lu et al., 2020; Sun et al., 2021; Huang and Wu, 2022). In this paper, the proposed SSCAC algorithm constructs a self-training subspace clustering framework based on the low-rank representation with distance penalty (LRRADP) (Fei et al., 2017) by using the high coordination between Gaussian fields and harmonic functions (GFHF) (Zhu et al., 2003) and low-rank representation.

In a semi-supervised learning framework, the dataset is usually formulated as ***X*** = [***x***_1_, ***x***_2_, …, ***x***_*l*_, ***x***_*l*+1_, …, ***x***_*n*_] = [***X***_*L*_, ***X***_*U*_] ∈ ***R*** ^*m*×*n*^, where ${\left.x_{i}\right|}_{i=1}^{l}$ and ${\left.x_{i}\right|}_{i=l+1}^{n}$ are labeled and unlabeled data, ***X***_*L*_ and ***X***_*U*_ are the labeled and unlabeled datasets, *c* is the number of clusters, the corresponding label set is ***L***_*a*_ = {1, …, *c*}, the label of datum ***x***_*i*_ is *y*_*i*_ ∈ ***L***_*a*_. In order to make different newly labeled data act on semi-supervised gene clustering with different strengths, this paper introduces label confidence to GFHF semi-supervised clustering, and the proposed SSCAC objective function is:

$$\frac{1}{2}\overset{n}{\underset{i,j=1}{\sum }}{\left\|F_{i}-F_{j}\right\|}^{2}W_{ij}+\lambda_{\infty }\overset{l}{\underset{i=1}{\sum }}{\left\|F_{i}-\mu_{i}Y_{i}\right\|}^{2}$$ (1)

where $F={\left[F_{1}^{T},\ldots ,F_{n}^{T}\right]}^{T}\in R^{n\times c}$ is the label prediction matrix, vector ***F***_*i*_ denotes the attribution of ***x***_*i*_ to each cluster; $Y={\left[Y_{1}^{T},\ldots ,Y_{n}^{T}\right]}^{T}\in B^{n\times c}$ is the binary label indication matrix, vector ***Y***_*i*_ corresponds to the label of ***x***_*i*_, if the label *y*_*i*_ = *k* then *Y*_*ik*_ = 1, otherwise *Y*_*ik*_ = 0; *λ*_*∞*_ is a very large constant; *μ*_*i*_ ∈ (0, 1] is the label confidence of the labeled datum ***x***_*i*_(*i* = 1, 2, …, *l*); *W*_*ij*_ is the element of the LRRADP affinity matrix ***W*** obtained by:

$$W=\left(Z+Z^{T}\right)/2$$ (2)

In LRRADP, ***Z*** ∈ ***R*** ^*n*×*n*^ is the low-rank representation matrix and ***Z***_*i*_ is the vector of coefficients of datum ***x***_*i*_ represented by other data; ***E*** ∈ ***R*** ^*m*×*n*^ is the noise matrix. The iterative update equations are as follows (Fei et al., 2017):

$$\begin{array}{c} Z_{p+1}=\arg\underset{Z}{min}{\left\|Z_{p}\right\|}_{*}+\frac{\beta_{p}}{2}\left({\left\|X-XZ_{p}-E_{p}+\Lambda_{1,p}/\beta_{p}\right\|}_{2}^{2}+\|Z_{p}-H_{p}+\Lambda_{2,p}/\beta_{p}{\|}_{2}^{2}\right) \end{array}$$ (3)

$$E_{p+1}=\arg\underset{E}{\min}{\left\|E_{p}\right\|}_{1}+\frac{\beta_{p}}{2}{\left\|X-XZ_{p}-E_{p}+\Lambda_{1,p}/\beta_{p}\right\|}_{2}^{2}$$ (4)

where ‖ ***Z*** ‖_*_ = *∑*_*i*_ *σ*_*i*_(***Z***) is the nuclear norm of ***Z***, which is used as a convex approximation of matrix rank, *σ*_*i*_(***Z***) denotes the *i*-th singular value of ***Z***; ‖.‖_1_ and ‖.‖_2_ are the *l*_1_-norm and *l*_2_-norm, respectively; auxiliary variable ***H***, Lagrange multipliers **Λ**_1_, **Λ**_2_ and penalty parameter *β* are determined by the following equations:

$$H_{p+1}=\arg\underset{H}{\min}\lambda_{2}tr\left(\Xi \left(D⊗H_{p}\right)\right)+\frac{\beta_{p}}{2}{\left\|Z_{p}-H_{p}+\Lambda_{2,p}/\beta_{p}\right\|}_{2}^{2}$$ (5)

$$\Lambda_{1,p+1}=\Lambda_{1,p}+\beta_{p}\left(X_{p+1}-X_{p+1}Z_{p+1}-E_{p+1}\right)$$ (6)

$$\Lambda_{2,p+1}=\Lambda_{2,p}+\beta_{p}\left(Z_{p+1}-H_{p+1}\right)$$ (7)

$$\beta_{p+1}=\min\left(\beta_{\max},\rho \beta_{p}\right)$$ (8)

In the update equations, *λ*_1_ > 0 and *λ*_2_ > 0 are balance parameters to trade off among the low-rank representation, noise and adaptive distance penalty.

In the SSCAC objective function defined by Eq. 1, the first term is the same as that of the original GFHF, which ensures the smoothness of data labels on the LRRADP graph. The second term is the label fitness term, which incorporates the label confidence *μ*_*i*_ and applies it to the label indication vector ***Y***_*i*_ of the labeled datum ***x***_*i*_. Actually, the objective function of GFHF is a special case of that of SSCAC with *μ*_*i*_ = 1 for each labeled datum ***x***_*i*_(*i* = 1, 2, …, *l*). That is, the SSCAC objective function is the extension of that of GHFH, which further considers the label confidences of the labeled data and can be applied to self-training clustering. Minimizing Eq. 1 can achieve both the manifold smoothness of the partition results in subspaces and the maximum matching between the predicted label and the label of labeled data under the effect of label confidence.

In the self-training process of SSCAC, newly labeled data are selected based on density-pointing relationships between data (Wu et al., 2018; Li et al., 2019) and added to the labeled dataset ***X***_*L*_ to guide the next iteration of self-training learning. The newly labeled data selection strategy will be detailed in the next Subsection. The rules for setting the label confidence *μ*_*i*_ in Eq. 1 are as follows: 1) if ***x***_*i*_ is an initially labeled datum, set the label confidence *μ*_*i*_ = 1 with complete confidence in its label accuracy; 2) if ***x***_*i*_ is a newly labeled datum of the current iteration of self-training, *μ*_*i*_ is initialized to a random number within (0,1], and then adaptively adjusted based on the semi-supervised learning value of ***x***_*i*_. The specific strategy is detailed in Section 2.3; 3) only the label confidences of the newly labeled data selected in the current iteration are adjusted, the adjusted confidences remain unchanged in the subsequent iterations of self-training.

The advantage of adding the label confidence in Eq. 1 is that the value can effectively regulate the supervision strength of newly labeled data on semi-supervised gene clustering, which improves the clustering accuracy on gene expression data. The analysis is as follows: 1) if the newly labeled datum ***x***_*i*_ is mislabeled, i.e., the position of 1 in the label indication vector ***Y***_*i*_ does not match that of the actual label, the label prediction vector ***F***_*i*_ will be predicted in the wrong direction under the effect of the second term of Eq. 1, and the larger the label confidence *μ*_*i*_, the larger the prediction bias. In the first term of Eq. 1, the elements corresponding to data in the same subspace in the LRRADP similarity matrix ***W*** are relatively large and those corresponding to data in different subspaces are small, so that labels are mainly propagated among data in the same subspace, then the mislabeled datum ***x***_*i*_ will lead to the label prediction bias of unlabeled data in the same gene clustering. Therefore, reducing the label confidence of mislabeled datum ***x***_*i*_ can effectively mitigate its negative impact on semi-supervised gene clustering; 2) if the newly labeled datum ***x***_*i*_ has correct label, the second term of Eq. 1 can guide ***F***_*i*_ to obtain correct prediction, and then realize correct label propagation for unlabeled data in the same subspace under the effect of the first term of Eq. 1. Obviously, increasing the label confidence of correctly labeled datum is beneficial to improve the partition accuracy of unlabeled data.

The matrix form of the SSCAC objective function is:

$$tr\left(F^{T}LF\right)+tr{\left(F-\mu ⊗Y\right)}^{T}U\left(F-\mu ⊗Y\right)$$ (9)

where ***L*** ∈ ***R*** ^*n*×*n*^ is the graph Laplacian matrix, ***L*** = ***D*** − ***W***, ***D*** is a diagonal matrix, *D*_*ii*_ = *∑*_*j*_ *W*_*i*,*j*_; ***U*** ∈ ***R*** ^*n*×*n*^ is also a diagonal matrix, the first *l* and the remaining *n* − *l* diagonal elements are *λ*_*∞*_ and 0, respectively; ⊗ denotes the Hadamard product; ***μ*** ∈ ***R*** ^*n*×*c*^, if the label of ***x***_*i*_(*i* = 1, 2, …, *l*) is *k*(*k* = 1, 2, …, *c*), then the *k*-th element in the *i*-th row vector is the label confidence of ***x***_*i*_, and all the other elements in the row vector are 0. For each unlabeled datum, all elements in the corresponding row vector are set to 0. By setting the derivative of Eq. 9 with respect to ***F*** to zero, the following equation can be easily obtained:

$$F={\left(L+U\right)}^{-1}U\mu Y$$ (10)

Then the predicted label of the unlabeled datum ***x***_*i*_ can be assigned by:

$${\hat{y}}_{i}=\arg\underset{k}{\max}F_{ik}$$ (11)

### 2.2 Newly labeled data selection strategy based on density relationships

In the self-training process, how to select newly labeled data from the unlabeled dataset ***X***_*U*_ and iteratively expand the labeled dataset ***X***_*L*_ is an important issue. Most self-training learning methods (Nie et al., 2012; Xia et al., 2018) rely entirely on the performance of learning models and ignore the potential density information in datasets. Relatively speaking, the strategy based on the data density relationships is not restricted by the distribution of initially labeled data and entire data space (Wu et al., 2018), and is more suitable for self-training learning on non-spherically distributed data.

In the self-training process of SSCAC, newly labeled data are selected based on density-pointing relationships between data (Wu et al., 2018; Li et al., 2019). The strategy utilizes clustering by fast search and find of density peaks (DPC) (Rodriguez and Laio, 2014), and for each datum ***x***_*i*_, its local density *ρ*_*i*_ can be defined as:

$$\rho_{i}=\underset{j}{\sum }\chi \left(d_{ij}-d_{c}\right),\;\chi \left(x\right)=\left\{\begin{array}{c} 1,\;x<0\;\\ 0,\;x\geq 0\; \end{array}\right.$$ (12)

where *d*_*ij*_ is the Euclidean distance between ***x***_*i*_ and ***x***_*j*_, and *d*_*c*_ is the cut-off distance. It can be seen that the value of local density *ρ*_*i*_ is the number of data whose distance from ***x***_*i*_ is less than *d*_*c*_. In addition, DPC defines the minimum distance between ***x***_*i*_ and other data with higher local densities as follows:

$$\delta_{i}=\left\{\begin{array}{c} \max_{j}\left(d_{ij}\right),\;\forall j,\;\rho_{i}\geq \rho_{j}\\ \min_{j:\rho_{i}<\rho_{j}}\left(d_{ij}\right),\text{ others} \end{array}\right.$$ (13)

The newly labeled data selection strategy calculates *ρ*_*i*_ and *δ*_*i*_ for each datum ***x***_*i*_ and make ***x***_*i*_ point to its nearest datum ***x***_*j*_ with a higher local density, then ***x***_*j*_ is called the “next” datum of ***x***_*i*_ and ***x***_*i*_ is the “previous” datum of ***x***_*j*_. Then, the strategy constructs the density-pointing relationships of low-density data to high-density data by selecting the “next” and “previous” unlabeled data of labeled data in batches and set their selection orders. Specifically, all the “next” data of data in the original labeled dataset ***X***_*L*_ are firstly selected from the unlabeled dataset ***X***_*U*_, and their selection orders are set to 1. That is, these data are viewed as the ones that should be labeled in the first iteration of self-training and used as the newly labeled data to expand the labeled dataset. In the next iteration, all the “next” data of the newly labeled data of the previous iteration are selected from ***X***_*U*_, and their selection orders increase by 1. This step repeats until there exists no “next” data of the newly labeled data of the previous iteration in ***X***_*U*_. If there are still unselected data in ***X***_*U*_, the selection orders of these remaining data can be set according to the “previous” relationships using the similar process. It can be seen that the unlabeled data with the same selection orders form the newly labeled dataset of the same iteration of self-training, on which basis the proposed SSCAC algorithm can expand the labeled dataset ***X***_*L*_ iteratively and realize self-training clustering.

### 2.3 Adaptive adjustment of label confidence based on gravitational search algorithm

According to the analysis of the SSCAC objective function in the previous subsection, it is obvious that the value of different newly labeled data should vary for semi-supervised learning. If the newly labeled datum ***x***_*i*_ is mislabeled, its incorrect label will propagate to the unlabeled data in the same subspace, making these data together with ***x***_*i*_ have significant differences in the label prediction vectors from those of the correctly labeled data in that subspace. In this case, Eq. 1 will inevitably result in a large function value, and ***x***_*i*_ can be regarded as a low-value newly labeled datum. Conversely, the newly labeled datum ***x***_*i*_ with correct label can propagate its correct label in the subspace it belongs to, so that the unlabeled data in this subspace will obtain similar label prediction vectors to those of the correctly labeled data. In this case, the objective function value of Eq. 1 will be relatively small, and ***x***_*i*_ can be regarded as a high-value newly labeled datum. Therefore, the SSCAC algorithm proposed in this paper measures the semi-supervised learning value of newly labeled data by the objective function value of Eq. 1, and on this basis, achieves the adaptive adjustment of label confidence.

Gravitational search algorithm (GSA) (Rashedi et al., 2009) is an optimization method based on the law of gravity, which is easy to implement and requires fewer parameters. It has been proven in the literature that GSA outperforms heuristic search algorithms such as PSO and GA (Mirjalili et al., 2012; Kumar et al., 2013). The search particles in GSA are a set of individuals that attract each other and generate motion in the solution space, the position of the individual is the solution of the optimization problem. Under the influence of gravity, the individuals move toward the individuals with heavier masses, which correspond to better solutions. To distinguish from the iterations of self-training learning, the iteration index of GSA is referred to as time in this paper. In the *r*-th iteration of self-training, let *I* be the number of newly labeled data, $X^{r}=\left(x_{1}^{r},x_{2}^{r},\ldots ,x_{i}^{r},\ldots ,x_{I}^{r}\right)$ be the set of newly labeled data, we use the label confidences of these newly labeled data to compose the label confidence vector. Specifically, the label confidence vector can be represented as the positions of particles when optimized by GSA, the position of GSA particle *a* at time *t* is defined by:

$$\mu_{a}^{r}\left(t\right)=\left(\mu_{1,a}\left(t\right),\mu_{2,a}\left(t\right),\ldots ,\mu_{i,a}\left(t\right),\ldots ,\mu_{I,a}\left(t\right)\right),\;a=1,2,\ldots ,N$$ (14)

where *N* is the population size, *μ*_*i*,*a*_(*t*) is the label confidence of the *i*-th newly labeled datum $x_{i}^{r}$ in particle *a* at time *t*, which is initialized to a random number within (0,1].

Based on the SSCAC objective function given in Eq. 9, the GSA fitness function of particle *a* at time *t* is defined as:

$${\mathrm{fi}tness}_{a}\left(t\right)=tr\left(F^{T}LF\right)+tr{\left(F-\mu_{a}^{r}\left(t\right)⊗Y\right)}^{T}U\left(F-\mu_{a}^{r}\left(t\right)⊗Y\right)$$ (15)

For the *i*-th newly labeled datum $x_{i}^{r}$ , the force acting on particle *a* from particle *b* at time *t* is expressed as:

$$f_{i,ab}\left(t\right)=G\left(t\right)\frac{M_{a}\left(t\right)\times M_{b}\left(t\right)}{R_{ab}\left(t\right)+\varepsilon }\left(\mu_{i,b}\left(t\right)-\mu_{i,a}\left(t\right)\right)$$ (16)

where *G*(*t*) is gravitational constant at time *t*, *M*_*a*_(*t*) and *M*_*b*_(*t*) are the inertial masses of particle *a* and particle *b*, *R*_*ab*_(*t*) is the Euclidean distance between particle *a* and particle *b*, and *ɛ* is a tiny constant to avoid zero denominator. The following equation can be used to determine the gravitational constant *G*(*t*):

$$G\left(t\right)=G_{0}e^{\left(-\alpha \frac{t}{T}\right)}$$ (17)

where *G*_0_ is the initial value of the gravitational coefficient, *α* is the decay coefficient, *G*_0_ and *α* are usually taken as 100 and 20 (Rashedi et al., 2009), and *T* is the maximum time.

During the motion of a particle, the inertial mass *M*_*a*_(*t*) of particle *a* can be updated according to the adapted value:

$$m_{a}\left(t\right)=\frac{{\mathrm{fi}tness}_{a}\left(t\right)-worst\left(t\right)}{best\left(t\right)-worst\left(t\right)}$$ (18)

$$M_{a}\left(t\right)=\frac{m_{a}\left(t\right)}{\sum_{b=1}^{N}m_{b}\left(t\right)}$$ (19)

where *m*_*a*_(*t*) is the intermediate variable, best(*t*) and worst(*t*) are the best and worst fitness values among all particles at time *t*, respectively. In this paper, the particle position that makes the fitness value Eq. 15 obtain the minimum value is selected as the label confidence of the newly labeled data. Here, best(*t*) and worst(*t*) are respectively given by:

$$best\left(t\right)=\underset{b\in \left\{1,\ldots ,N\right\}}{\min}{\mathrm{fi}tness}_{b}\left(t\right)$$ (20)

$$worst\left(t\right)=\underset{b\in \left\{1,\ldots ,N\right\}}{\max}{\mathrm{fi}tness}_{b}\left(t\right)$$ (21)

According to Newtonian gravity and the laws of motion, the gravitational force on particle *a* in the *i*-th dimension at time *t* is the sum of the gravitational forces from all other particles.

$$f_{i,a}\left(t\right)=\overset{N}{\underset{b=1,b\neq a}{\sum }}{rand}_{b}f_{i,ab}\left(t\right)$$ (22)

where rand_*b*_ is a random number within [0,1]. According to Newton’s second law, the acceleration of particle *a* in the *i*-th dimension is:

$$s_{i,a}\left(t\right)=\frac{f_{i,a}\left(t\right)}{M_{a}\left(t\right)}$$ (23)

Therefore, the velocity and position of particle *a* in the *i*-th dimension at the next time are updated by:

$$\left\{\begin{array}{c} v_{i,a}\left(t+1\right)={rand}_{a}\times v_{i,a}\left(t\right)+s_{i,a}\left(t\right)\\ \mu_{i,a}\left(t+1\right)=\mu_{i,a}\left(t\right)+v_{i,a}\left(t+1\right) \end{array}\right.$$ (24)

where rand_*a*_ is a random number within [0,1], the initial velocity *v*_*i*,*a*_(0) is 0.

When time *t* reaches *T*, the position of the particle that obtains the minimum fitness value is used as the label confidence vector ***μ*** ^*r*^ for the newly labeled data ***X*** ^*r*^ at the *r*-th iteration of self-training. Then, we can update ***μ***, ***U*** and ***Y*** in Eq. 9 based on the obtained label confidence vector ***μ*** ^*r*^, the newly labeled data ***X*** ^*r*^, their predicted labels respectively, and guide the subsequent iterations of self-training. It can be seen that the proposed strategy can adaptively adjust the label confidence based on the semi-supervised learning value of the newly labeled data. By reducing the label confidences of low-value newly labeled data, we can effectively reduce their effect on semi-supervised learning and thus alleviate the problem of mistaken reinforcement in the self-training gene clustering.

### 2.4 The procedure of the proposed SSCAC algorithm

**FIGURE 1:**
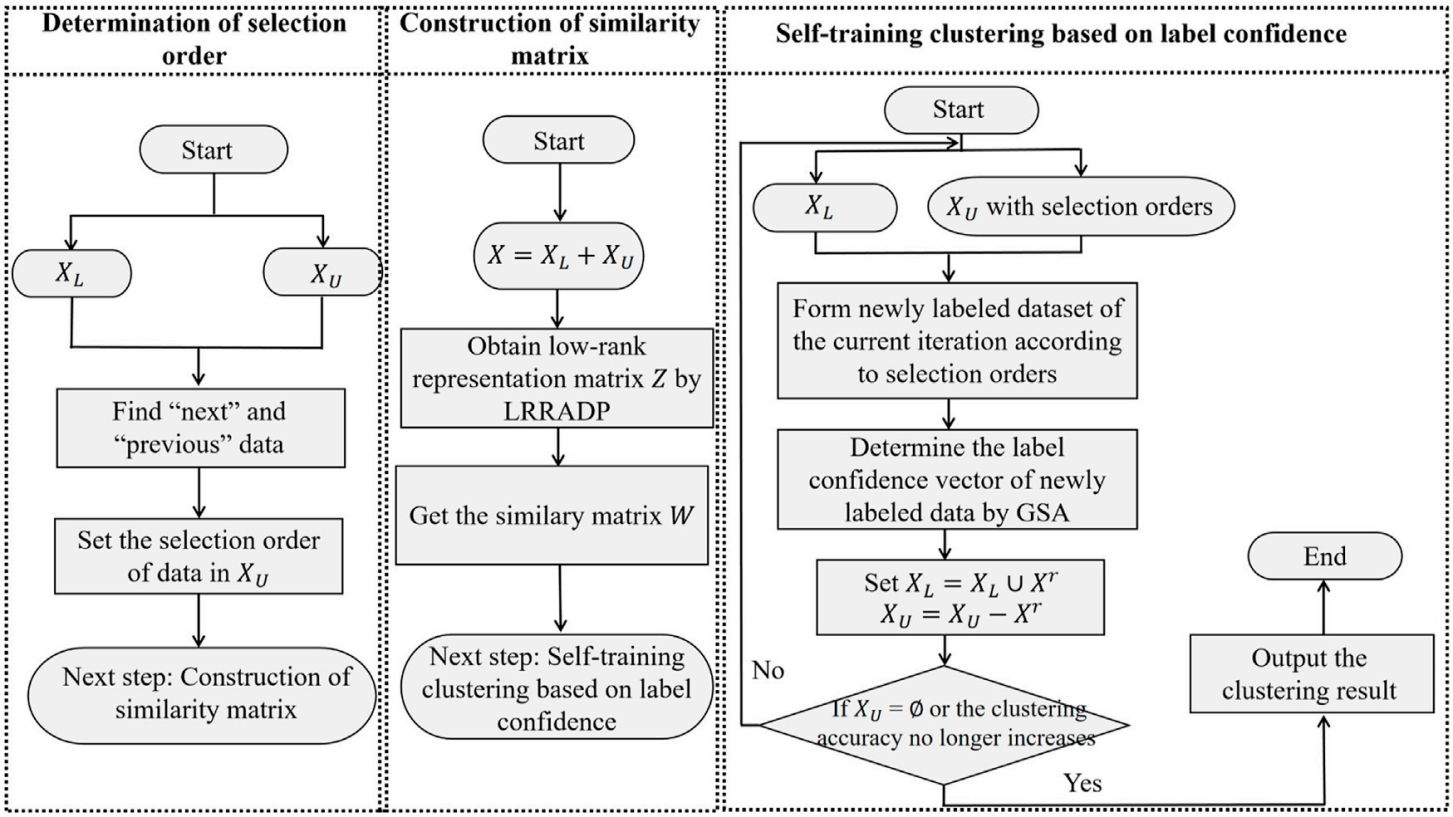
A framework of SSCAC.

For a set of gene expression data ***X*** = [***x***_1_, ***x***_2_, …, ***x***_*l*_, ***x***_*l*+1_, …, ***x***_*n*_] = [***X***_*L*_, ***X***_*U*_] ∈ ***R*** ^*m*×*n*^, the detailed procedure of SSCAC is given in Algorithm 1, and the framework of SSCAC is shown in Figure 1. In SSCAC, the stopping condition is set to ***X***_*U*_ = ∅ or the clustering accuracy no longer increases as suggested in the literature (Qu et al., 2019).

Algorithm 1 Note that when the stopping condition is that the clustering accuracy no longer increases, the labels of the remaining data in ***X***_*U*_ are obtained based on ***F***.

Step 1: Set the parameters, including maximum value of penalty parameter *β*_*max*_, iteration stop parameter *ξ*, constant *ρ*, balance parameters *λ*_1_ and *λ*_2_ of the LRRADP algorithm, and population size *N*, maximum time *T*, constant *ɛ* of the GSA algorithm.
Step 2: For each datum ***x***_*i*_ in ***X***, initialize its selection order ***O*** (*i*) = 0, calculate *ρ*_*i*_, *δ*_*i*_ according to Eqs 12, 13, and find the “next” and “previous” data of ***x***_*i*_ based on *ρ*_*i*_, *δ*_*i*_. Set the iteration index of the unlabeled data selection *r* = 1, then set the selection order of unlabeled data by the following steps.
  1) For each datum ***x***_*i*_ in ***X***_*U*_, if ***x***_*i*_ is the “next” datum of a datum in ***X***_*L*_, set its selection order ***O*** (*i*) = *r*.
  2) Set *r* = *r* + 1. For each unselected datum ***x***_*i*_ in ***X***_*U*_, if ***x***_*i*_ is the “next” datum of a datum whose selection order is *r* − 1, set ***O*** (*i*) = *r*.
  3) If there still exists “next” data of data whose selection orders are *r* in ***X***_*U*_, then return to 2); otherwise, set *r* = *r* + 1 and go to 4).
  4) For each unselected datum ***x***_*i*_ in ***X***_*U*_, if ***x***_*i*_ is the “previous” datum of the selected data, then set ***O*** (*i*) = *r*.
  5) Set *r* = *r* + 1. For each unselected datum ***x***_*i*_ in ***X***_*U*_, if ***x***_*i*_ is the “previous” datum of a datum whose selection order is *r* − 1, set ***O*** (*i*) = *r*.
  6) If there still exists “previous” data of data whose selection orders are *r* in ***X***_*U*_, then return to 5); otherwise, get the vector ***O*** of selection order for unlabeled data and go to Step3.
Step 3: Initialize ***Z*** = ***H*** = ***E*** = **Λ**_1_ = **Λ**_2_ = 0, *β*_0_ = 1. Set the iteration index of the LRRADP algotirhm *p* = 0, calculate Eqs 3–8 iteratively until $\left\|Z_{p+1}-Z_{p}\right\|/\left\|Z_{p}\right\|\geq \xi$ to obtain the low-rank representation matrix ***Z*** of ***X***, and get the similarity matrix ***W*** = (***Z*** + ***Z*** ^*T*^)/2. Set the iteration index of self-training *r* = 1, initialize ***U*** and ***Y*** based on initial ***X***_*L*_, set label confidence ***μ***_*i*_ = 1 for each datum in ***X***_*L*_, get initial predicted labels according to Eqs 10, 11.
Step 4: For the *r*-th iteration of self-training, initialize the newly labeled dataset ***X*** ^*r*^ = ∅. For each datum ***x***_*i*_ whose ***O*** (*i*) = *r*, label ***x***_*i*_ according to its predicted label ${\hat{y}}_{i}$ , set $X^{r}=X^{r}\cup \left\{x_{i}\right\}$ .
Step 5: Determine the label confidence vector ***μ*** ^*r*^ for the newly labeled data ***X*** ^*r*^ by the following steps.
  1) For each particle *a*(1 ≤ *a* ≤ *N*), randomly generate each element of its initial position $\mu_{a}^{r}\left(0\right)$ within (0,1]. Set the particle search time *t* = 1.
  2) For each particle *a*, calculate its fitness value at time *t* according to Eq. 15, update its position $\mu_{a}^{r}\left(t\right)$ according to Eqs 16–24.
  3) If *t* < *T*, then set *t* = *t* + 1 and return to 2); otherwise, the position of the particle with minimum fitness value is used as the label confidence vector ***μ*** ^*r*^ and go to Step 6.
Step 6: Set ***X***_*L*_ = ***X***_*L*_ ∪ ***X*** ^*r*^, ***X***_*U*_ = ***X***_*U*_ − ***X*** ^*r*^, update ***μ***, ***U*** and ***Y***. Update the label prediction matrix ***F*** and predicted labels of the data according to Eqs 10, 11. If ***X***_*U*_ = ∅ or the clustering accuracy no longer increases compared with the previous iteration, stop and output the clustering result; otherwise, set *r* = *r* + 1 and return to Step 4.

## 3 Experimental results and analysis

### 3.1 Experimental setup

In this paper, comparative experiments are conducted in two benchmark gene expression datasets, as shown in Table 1. The Gal dataset (Ideker et al., 2001) is composed of gene expression measurements for 205 genes involved in galactose use in *Saccharomyces cerevisiae*. The gene expression profiles were measured with four replicate assays across 20 time points and the expression patterns reflect four functional categories. Yeast is a UCI dataset, which aims to predict the localization sites of proteins in cells and contains 1,484 yeast genes with eight methods of predicting protein localization sites in dimensions. Besides, we also demonstrate the applications of the proposed algorithm in other datasets, details of the datasets are tabulated in Supplementary Table S1, and the clustering results can be seen in Supplementary Tables S2, S3.

**TABLE 1:** The description of experimental datasets.

Index	Datesets	Types	Number of genes(*n*)	Number of features(*m*)	Classes(*c*)
1	Gal	Gene expression	205	80	4
2	Yeast	Gene expression	1,484	8	10

To verify the effectiveness of the SSCAC algorithm proposed in this paper for gene expression data, SSCAC is compared with three unsupervised clustering algorithms and four semi-supervised learning algorithms, including the K-means clustering based on the original gene expression data ***X***, the K-means clustering based on the low-rank representation matrix ***Z*** (LRR + Kmeans) (Xia et al., 2018), the NCut clustering based on the LRR similarity matrix ***W*** (LRR + NCut) (Liu et al., 2013), SSC-LRR (Xia et al., 2018), STDP (Wu et al., 2018), STDPNF (Li et al., 2019), and LRRADP + GFHF (Fei et al., 2017) algorithms, where SSC-LRR, STDP, and STDPNF are self-training methods. To illustrate the effectiveness of the filter in the STDPNF algorithm, both STDP and STDPNF use KNN as the base classifier. Based on the suggestion of the literature (Xia et al., 2018), the balance parameter *λ* in LRR and SSC-LRR algorithms is tuned within [2^−3^, 2^4^], and the parameter value corresponding to the optimal clustering result is selected, so we set *λ* = 0.1 for all the datasets. In LRRADP + GFHF and SSCAC, we set the balance parameters *λ*_1_ = 100, *λ*_2_ = 1 and *λ*_*∞*_ = 1 × 10^5^, and the maximum value of penalty parameter *β*_max_ = 10^4^, iteration stop parameter *ξ* = 10^−5^, constant *ρ* = 1.01. And we set the maximum time of the adaptive adjustment of label confidence *T* = 100, population size *N* = 50, and constant *ɛ* = 2.2204e − 16 in SSCAC, the cut-off distance *d*_*c*_ is the corresponding value of data distance sorted in ascending order of 2%, and the other parameters in comparison methods are set as suggested in the original studies. Similar to literature (Nie et al., 2012; Fei et al., 2017), the experiments in this paper form the initial labeled dataset ***X***_*L*_ by randomly selecting 10% of the data in each dataset, and the rest of the data form the unlabeled dataset ***X***_*U*_. All algorithms are run 10 times with randomly selected initial labeled data, and the algorithm performance is evaluated using the mean value of the results.

### 3.2 Evaluation metrics

To assess the partition performance, we use two popular metrics, accuracy (ACC) and Normalized mutual information (NMI).

(1) ACC is calculated by

$$ACC=\frac{\sum_{i=1}^{n}\delta \left(y_{i},map\left({\hat{y}}_{i}\right)\right)}{n}$$ (25)

where *y*_*i*_ and ${\hat{y}}_{i}$ denote the true label and predicted label of ***x***_*i*_, respectively, $map\left({\hat{y}}_{i}\right)$ denotes the mapping match between the true label and the predicted label, and $\delta \left(y_{i},map\left({\hat{y}}_{i}\right)\right)=1$ when $y_{i}=map\left({\hat{y}}_{i}\right)$ , otherwise, it is 0. The closer the value of ACC is to 1, the higher the partition accuracy is.

(2) NMI is calculated by

$$NMI=\frac{2I\left(A,B\right)}{H\left(A\right)+H\left(B\right)}$$ (26)

where *A* and *B* denote the vectors consisting of the true and predicted labels corresponding to the partition results, respectively. *I*(*A*, *B*) denotes the mutual information measure, *H*(*A*) and *H*(*B*) denote the entropy of *A* and *B*, respectively. The value of *NMI* is between 0 and 1, and a larger value of *NMI* indicates a better partition performance.

### 3.3 Comparative results and analysis

Table 2 shows the ACC and NMI results of eight algorithms on two benchmark gene expression datasets. The optimal and suboptimal results are marked with bold and italics, respectively.

**TABLE 2:** ACC and NMI results of each algorithm on two benchmark gene expression datasets.

Datasets	Evaluation metrics	K-means	LRR + Kmeans	LRR + NCut	SSC−LRR	STDP	STDPNF	LRRADP + GFHF	SSCAC
Gal	ACC	0.8517	0.8639	0.8585	0.8912	0.9059	0.9024	*0.9205*	**0.9371**
	NMI	0.8006	0.8043	0.7493	*0.8079*	0.8014	0.7858	0.7562	**0.8113**
Yeast	ACC	0.3647	0.3726	0.3760	0.3261	0.4816	0.4387	*0.4926*	**0.4987**
	NMI	0.2652	0.2543	0.1421	0.2024	0.2708	*0.2781*	0.2715	**0.2782**

Bold and italic values indicates the optimal value and suboptimal values.

From the results in Table 2, it can be seen that.

(1) For the two benchmark gene expression datasets, the clustering results of the SSCAC algorithm proposed in this paper are significantly better than those of the comparison algorithms, indicating the effectiveness of the proposed self-training subspace clustering framework and the adaptive adjustment strategy of label confidence. In addition, the performance of the semi-supervised learning methods outperforms that of the unsupervised clustering algorithms in general, reflecting the advantages of the semi-supervised learning methods.
(2) Among the unsupervised clustering algorithms, LRR + Kmeans and LRR + NCut perform better overall than the K-means algorithm based on the original gene expression data ***X***. Compared with K-means, LRR + Kmeans and LRR + NCut improve ACC by an average of 1.80% and 1.95% for two benchmark gene expression datasets. This is because the low-rank representation matrix ***Z*** and the similarity matrix ***W*** can better reflect the properties of the gene expression data in the low-dimensional subspace, thus more discriminative features can be extracted from the data (Xia et al., 2018). Compared with LRR, the LRRADP used in the proposed SSCAC algorithm further enhances the locality of the model and can better capture the subspace structure of gene expression data. This advantage of SSCAC will be further demonstrated and analyzed in Section 3.7.
(3) Compared with the self-training algorithms SSC-LRR, STDP, and STDPNF, the SSCAC algorithm proposed in this paper has significant advantages. One of the main reasons is that the compared self-training methods implicitly assume that all newly labeled data have the same label confidence. As pointed out in the literature (Mellor et al., 2015; Xia et al., 2018; Li et al., 2019), the problem of mislabeling is inevitable, so setting the same label confidence for both mislabeled and correctly labeled data will lead to continuous reinforcement of incorrect labels during label propagation. Besides, the proposed SSCAC algorithm also outperforms the semi-supervised LRRADP + GFHF, the analysis and comparison will be detailed in the following ablation study.

**FIGURE 2:**
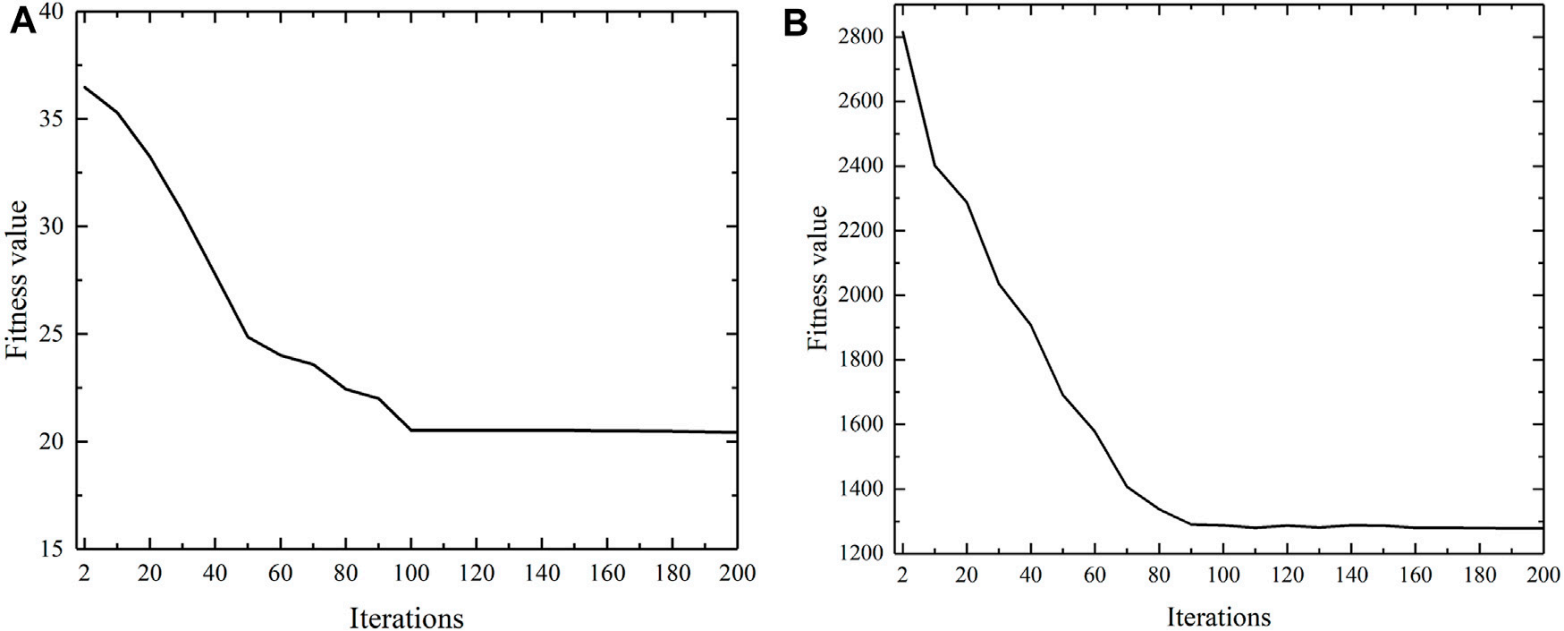
Convergence analysis of proposed updating strategy of confidence vector in SSCAC. **(A)** Gal; **(B)** Yeast.

In order to verify the convergence of the proposed updating strategy of confidence vector in SSCAC, convergence analysis experiments regarding the number of iterations versus fitness value are done for two benchmark gene expression datasets, Gal and Yeast. As shown in Figure 2, the fitness values flatten out with increasing iteration number and finally converge in approximately 100 iterations. Then, the position of the particle that obtains the minimum fitness value is used as the label confidence vector for the newly labeled data, on which basis SSCAC yields superior clustering results.

### 3.4 Ablation study

In order to validate the effectiveness of label confidence, we also conduct an ablation study. The ablation algorithm is referred to as SSCNAC, i.e., SSCAC without label confidence. In SSCNAC, the same label confidence *μ*_*i*_ = 1 is implicitly set for each newly labeled datum ***x***_*i*_ in the self-training process, thus SSCNAC is a self-training subspace clustering algorithm based on original GFHF. The parameter setting of SSCNAC is the same as that of SSCAC, and the performance of SSCNAC and SSCAC in terms of ACC and NMI is reported in Table 3. The optimal values of Table 3 are shown in bold. From Table 3, it can be seen that the proposed SSCAC algorithm achieves better clustering performance over SSCNAC. As with other self-training algorithms, SSCNAC performs self-training with complete confidence in the label accuracy of newly labeled data, and therefore suffers from the problem of mislabeling. Comparatively speaking, the proposed SSCAC algorithm introduces label confidences into the semi-supervised clustering objective function and adaptively adjusts them based on semi-supervised learning values, thus can effectively mitigate the negative impact of mislabeled data on self-training learning. This advantage of SSCAC will be further demonstrated in Table 4.

**TABLE 3:** Comparison of ACC and NMI of SSCNAC and SSCAC on two benchmark gene expression datasets.

Datasets	Evaluation metrics	SSCNAC	SSCAC
Gal	ACC	0.9361	**0.9371**
	NMI	**0.8153**	0.8113
Yeast	ACC	0.4966	**0.4987**
	NMI	0.2761	**0.2782**

Bold values indicates the optimal value.

**TABLE 4:** The newly labeled data selected in the last three iterations of SSCAC on Gal.

Iterations of self-training	Newly labeled data	Real labels	Predicted labels	Label confidences
7	***x***_23_	3	3	0.0090
7	***x***_65_	3	3	1.0000
7	***x***_92_	2	1	0.1821
7	***x***_101_	1	1	0.7275
7	***x***_108_	2	1	0.1990
7	***x***_128_	4	4	1.0000
7	***x***_152_	3	3	1.0000
7	***x***_165_	1	1	0.9626
8	***x***_24_	1	1	0.8331
8	***x***_40_	1	1	0.1622
8	***x***_99_	1	1	0.7804
8	***x***_163_	1	1	0.8924
9	***x***_30_	3	3	0.6928
9	***x***_183_	1	1	0.8974

Moreover, from Tables 2, 3, we can also observe that the clustering results of SSCNAC outperform those of LRRADP + GFHF, with an average improvement of 1.25% and 4.75% in ACC and NMI, respectively. In essence, the SSCNAC algorithm with fixed-label confidence is a direct extension of LRRADP + GFHF on self-training, which gives SSCNAC the ability to learn from unlabeled data in self-training framework and therefore has better generalization performance. The above results demonstrate the positive role of unlabeled data in self-training learning and the effectiveness of the proposed self-training subspace clustering framework based on GFHF for gene expression data.

### 3.5 Analysis of hyper-parameters

**FIGURE 3:**
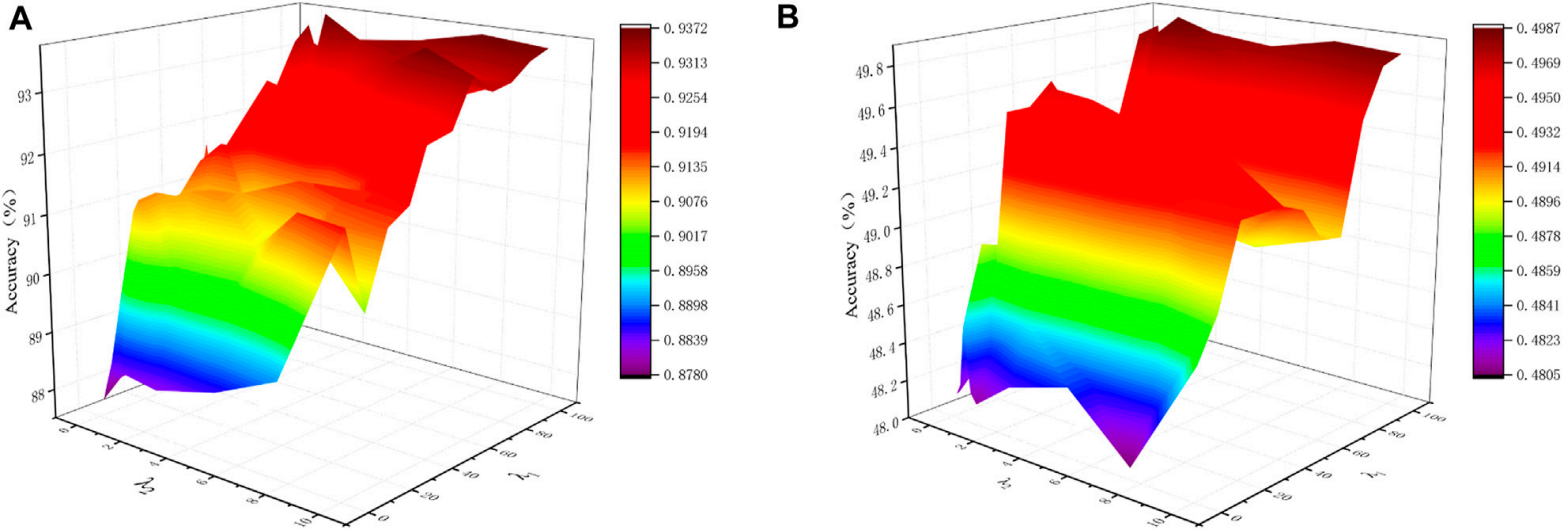
Impact analysis of the hyper-parameters on the performance of SSCAC. **(A)** Gal; **(B)** Yeast.

In the proposed SSCAC algorithm, *λ*_1_ and *λ*_2_ are balance parameters to trade off among the low-rank representation, noise and adaptive distance penalty. Figure 3 shows the impact of the two hyper-parameters on the performace of SSCAC. As can be observed, the proposed SSCAC algorithm is comparatively unaffected by hyper-parameters that are close to the ideal. To be more precise, we advise setting *λ*_1_ = 100 and *λ*_2_ = 1.

### 3.6 Analysis of the impact of initially labeled data ratio

**FIGURE 4:**
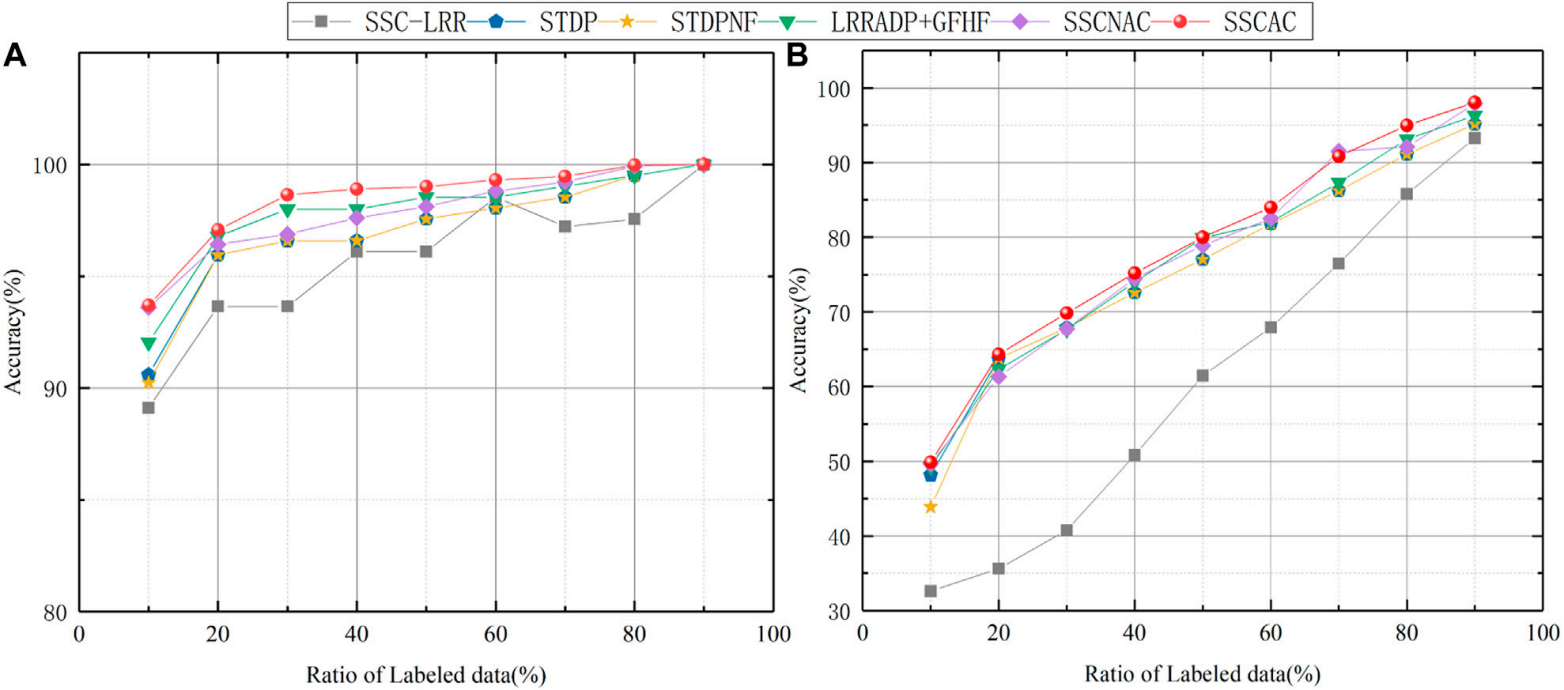
ACC of each algorithm with different ratio of initially labeled data. **(A)** Gal; **(B)** Yeast.

In order to analyze the impact of initially labeled data size on algorithm performance, we increase the initially labeled data ratio from 10% to 90% and conducted experiments, all algorithms are run 10 times. The average ACC curves of semi-supervised SSC-LRR, STDP, STDPNF, LRRADP + GFHF, SSCNAC and SSCAC algorithms are given in Figure 4.

It can be seen from Figure 4 that, in general, the partition accuracy of each algorithm increases along with the size of initially labeled data, the reason is that the increase in available label information helps to obtain models that fit the data more closely. It can also be seen from Figure 4 that in all cases, the ACC values of the proposed SSCAC algorithm are higher than those of the comparison algorithms, and this advantage of SSCAC is more evident in the cases when the initially labeled data ratios are relatively low. This is because that in such cases, the newly labeled data occupies a larger proportion of the labeled dataset and therefore plays a dominant role in the self-training process. Thus, the adaptive adjustment strategy of label confidence of SSCAC can reduce the influence of mislabeled data to a greater extent. As the proportion of initially labeled data increases, the dominant role of the newly labeled data in the self-training process decreases, and the semi-supervised learning performance of each algorithm tends to be similar. The above results and analysis indicate that the SSCAC algorithm proposed in this paper is more suitable for solving the semi-supervised clustering problem with less initially labeled data.

### 3.7 Analysis of the contribution of each part of the proposed SSCAC model

**FIGURE 5:**
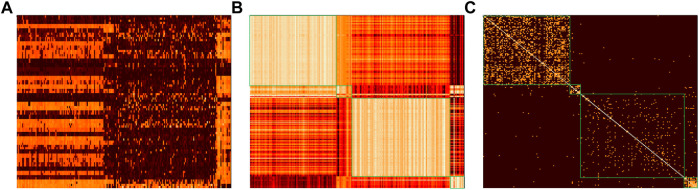
Visualization of original gene expression data and low-rank representation matrices on Gal. **(A)** Image of original data matrix ***X***; **(B)** Image of LRR low-rank representation matrix ***Z***; **(C)** Image of LRRADP low-rank representation matrix ***Z***.

In this section, we discuss the contribution of each part of the proposed model. The SSCAC model described by Eq. 9 consists of two parts: $tr\left(F^{T}LF\right)$ and tr(***F*** −***μY***)^*T*^ ***U*** (***F*** − ***μY***), which together make the model have high clustering accuracy. $tr\left(F^{T}LF\right)$ is the manifold smoothness term of the objective function, the LRRADP low-rank representation matrix ***Z*** adopted in SSCAC can effectively enhance the sparsity of the similarity matrix ***W*** and improve the discriminative property of gene expression data, which can then improve the clustering accuracy through the graph Laplacian matrix ***L***. To illustrate the advantage of the LRRADP low-rank representation, visualization of the original data matrix and the low-rank representation matrixs of LRR and LRRADP are plotted on the Gal dataset, as shown in Figure 5, and the data in each subplot are sorted according to their cluster labels in an ascending order. As seen from Figure 5, the low-rank representation matrix ***Z*** in both Figures 5B, C has a block-diagonal structure, i.e., the four high pixel rectangles along the diagonal of ***Z*** correspond to the four Gal gene clusters, respectively. It is obvious that compared with the original data matrix ***X***, the low-rank representation matrix ***Z*** can better reveal the subspace structure of gene expression data, i.e., the block-diagonal structure. Comparing Figures 5B, C, it can be seen that since LRRADP considers the locality of gene expression data while focusing on the global low-rank constraint, the resulting low-rank representation matrix ***Z*** is more sparse and the diagonal-block structure is more obvious, and thus can provide more discriminative information for SSCAC.

On the other hand, the second term of the SSCAC model, tr(***F*** − ***μY***)^*T*^ ***U*** (***F*** − ***μY***), incorporates the label confidence ***μ*** with the aim to reduce the label confidences of mislabeled data through the proposed adaptive adjustment strategy of label confidence, and mitigate their negative impact during the self-training iterations. In order to verify the effectiveness of the adaptive adjustment strategy of label confidence in the SSCAC model, we focus on the newly labeled data, as well as their real labels, predicted labels, and label confidences during the self-training process. In our experiments, all algorithms are run 10 times with randomly selected initial labeled data. Thus, the newly labeled data selected during the iteration of SSCAC are different for different initial labeled data. Here, we take one case of random selection of initial labeled data on GAL as an example, where SSCAC achieves convergence in nine iterations. The newly labeled data selected in the last three iterations and their label confidences are reported in Table 4, and similar results can be obtained for other iterations.

As seen from Table 4, the adaptive adjustment strategy proposed in this paper can effectively reduce the label confidences of the mislabeled data, such as ***x***_92_ and ***x***_108_ in the 7-th iteration, and assign large confidences to the correctly labeled data. From Table 4, we can also observe that the label confidence of the correctly labeled datum ***x***_23_ is rather small. As pointed out in the literature (Chen et al., 2011), even though some datum has correct label, it may have less impact on supervised learning due to its low partition uncertainty. Therefore, it is reasonable to assign a lower confidence to such correctly labeled datum. Compared with the existing self-training methods that do not consider the label confidence of newly labeled data, SSCAC can adaptively adjust the strength of supervisory guidance for different newly labeled data in the self-training process and effectively mitigate the negative impact of mislabeled data, which helps to significantly improve the clustering accuracy on gene expression data.

## 4 Conclusion

To deal with the widely existing problem of mislabeling in self-training learning tasks, a novel self-training subspace clustering algorithm for gene clustering is proposed in this paper. In particular, label confidences are integrated into the self-training clustering model, and the corresponding determination strategy of label confidences is proposed to adaptively adjust the supervision strength of newly labeled data according to their semi-supervised learning values. Moreover, the low-rank representation with distance penalty is adopted to improve discriminative property of gene expression data. Compared with other state-of-the-art unsupervised and semi-supervised learning algorithms, the proposed SSCAC algorithm can effectively mitigate the negative impact of mislabeling and improve the stability and accuracy of gene clustering. In our future work, we will consider biological knowledge such as Gene Ontology annotation information, and extend the proposed model to multi-view clustering framework to further improve clustering performance on gene expression data.

## Data Availability

Publicly available datasets were analyzed in this study. This data can be found here: https://archive.ics.uci.edu/ml/datasets/Yeast, http://genomebiology.com/2003/4/5/R34.
