# Supplementary material for: A self-training subspace clustering algorithm based on adaptive confidence for gene expression data
Source: Front Genet. 2023 Mar 21;14:1132370. doi: 10.3389/fgene.2023.1132370 (PMC10070828; doi:10.3389/fgene.2023.1132370)
Supplement: Supplementary file 1 [file DataSheet1.PDF]

frontiers

Supplementary Material

1 SUPPLEMENTARY DATA

To further verify the generalization of the proposed SSCAC algorithm, we also demonstrate the applications of the proposed algorithm in other datasets, the details of the datasets are shown in Table S1, and the experimental results are shown in Table S2. Table S3 shows the comparison results of SSCAC with its ablation experiments. From the experimental results, we can see that the proposed SSCAC algorithm outperforms state-of-the-art methods on various datasets.

Table S1. The description of experimental datasets

Index	Datesets	Types	Samples(n)	Attributes(m)	Classes(c)
1	Glass	UCI	214	10	7
2	Ecoli	UCI	336	7	8
3	Cars	UCI	1728	6	4
4	Sonar	UCI	208	60	2
5	UKM	UCI	403	5	5
6	Ionosphere	UCI	351	6	2
7	COIL20	Images	1440	1024	20
8	YaleB	Images	2414	1024	30

Table S2. ACC and NMI results of each algorithm on eight datasets

Datasets	Evaluation Metrics	K-means	LRR+ Kmeans	LRR+NCut	SSC−LRR	STDP	STDPNF	LRRADP +GFHF	SSCAC
Glass	ACC	0.4822	0.4827	0.5374	0.4953	0.5474	0.5563	0.6257	**0.6294**
	NMI	0.3539	0.3616	0.3359	0.2927	0.3077	0.3080	0.3845	**0.4084**
Ecoli	ACC	0.5134	0.5833	0.5268	0.5461	0.8124	0.8144	0.8036	**0.8187**
	NMI	0.5819	0.5642	0.5213	0.4684	0.6536	**0.6568**	0.6142	0.6338
Cars	ACC	0.3240	0.3442	0.6152	0.3692	0.7203	0.7241	0.7448	**0.8154**
	NMI	0.0541	0.0807	0.1525	0.1088	0.2706	0.2668	0.3479	**0.4274**
Sonar	ACC	0.5433	0.5577	0.5644	0.5861	0.5894	0.5822	0.7269	**0.7447**
	NMI	0.0075	0.0124	0.0254	0.0234	0.0380	0.0339	0.2015	**0.2322**
UKM	ACC	0.4831	0.5511	0.3226	0.4381	0.6715	0.6890	0.6754	**0.7035**
	NMI	0.2964	0.2871	0.0597	0.1173	0.4120	0.4432	0.4508	**0.5066**
Ionosphere	ACC	0.7105	0.7114	0.5812	0.6182	0.7330	0.7203	0.7350	**0.7559**
	NMI	0.1332	0.1354	0.0019	0.0186	0.1530	0.1318	0.2042	**0.2404**
COIL20	ACC	0.5639	0.5706	0.6361	0.7194	0.8595	0.8560	0.9209	**0.9486**
	NMI	0.7433	0.7427	0.7365	0.7771	0.8735	0.8759	0.9488	**0.9649**
YaleB	ACC	0.0918	0.3796	0.7627	0.4453	0.2981	0.2630	0.9345	**0.9455**
	NMI	0.1298	0.5578	0.8289	0.5406	0.3674	0.3254	0.9153	**0.9189**

Table S3. Comparison of ACC and NMI of SSCNAC and SSCAC on eight datasets

Method	Evaluation Metrics	Glass	Ecoli	Cars	Sonar	UKM	Ionosphere	COIL20	YaleB
SSCNAC	ACC	**0.6327**	0.8107	0.8032	**0.7466**	0.6849	0.7407	0.9418	0.9450
	NMI	0.3990	0.6281	0.4199	0.2251	0.4741	0.2128	0.9608	0.9178
SSCAC	ACC	0.6294	**0.8187**	**0.8154**	0.7447	**0.7035**	**0.7559**	**0.9486**	**0.9455**
	NMI	**0.4084**	**0.6338**	**0.4274**	**0.2322**	**0.5066**	**0.2404**	**0.9649**	**0.9189**
